# *Cyperus esculentus* var. *sativus* Adapts to Multiple Heavy Metal Stresses Through the Assembly of Endophytic Microbial Communities

**DOI:** 10.3390/biology14010083

**Published:** 2025-01-16

**Authors:** Qiaofeng Liu, Jialing Lai, Yaozhong Zhang, Xin Wang

**Affiliations:** Department of Pathology and Pathophysiology, Chengdu Medical College, Chengdu 610083, China; laijialing2000@163.com (J.L.); center666@163.com (Y.Z.)

**Keywords:** endophytes, heavy metal, plants, microbial diversity, community function

## Abstract

Interactions between plants and their endophytes alter their metabolic functions and ability to cope with abiotic stresses. In this study, high-throughput sequencing was used to analyze the species diversity and functions of endophytes in *Cyperus esculentus* var. *sativus* (CES) tubers under different heavy metal stress conditions. The results indicated that the number of observed endophytic species in the tubers increased under heavy metal stress (*p* < 0.05), leading to changes in species diversity and composition. The response of tuber endophytes to different metal concentrations varied, with certain endophytic bacteria and fungi, such as *Pseudomonas*, *Novosphingobium*, and *Fusarium*, showing increased abundance and becoming the dominant species in the tubers. Additionally, new endophytic genera, *Actinophytocola* and *Monosporascus*, emerged at specific metal concentrations (*p* < 0.05). Fatty acid salvage was enriched in the endophytes of CES, which may play an important role in assisting CES in responding to multiple heavy metal stresses. These findings showed that CES tuber endophytes undergo adaptive changes to support the ability of plants to cope with heavy metal stress.

## Simple Summary

The study investigated the changes in the endophytic microbial community of *Cyperus esculentus* var. *sativus* (CES), a versatile plant rich in nutrients, under different heavy metal stress conditions using high-throughput sequencing. The results showed that the number and diversity of endophytic species in CES tubers increased under heavy metal stress, with specific microbial species becoming dominant. These changes helped CES better adapt to heavy metal stress, suggesting a potential role of endophytes in plant heavy metal tolerance.

## 1. Introduction

*Cyperus esculentus* var. *sativus* (CES) is rich in oil, starch, sugar, proteins, vitamins, and many minerals, making it a versatile plant for edible and medicinal purposes [1]. Extracts of CES have also been shown to have antioxidant, anti-inflammatory, and antiapoptotic properties [2]. CES can be grown in a wide range of polluted environments, and several studies have reported that it can be used for phytoremediation in polluted environments, including those associated with heavy metal pollution and diesel pollution [3,4,5]. In the industrial era, metals have become the main pollutants in soil. Metals are divided into essential and nonessential metals on the basis of their biological needs. Essential metals are vital elements for the growth and development of organisms. However, when the concentration of essential metals in an organism exceeds a certain level, the metals are toxic to the organism [6]. Nonessential metals enter organisms through pathways similar to those of essential metals. They can be toxic at very low doses [7]. Because of their nondegradable characteristics, metals can persist in the environment for a long time and continue to cause harm to organisms in the environment [8,9,10]. There is no single metal species in the same environment [11], with Cu, Zn, and Cd being the three most common metals found in most environments [12,13,14]. One study examined the metal content in soils from industrial areas and reported that the levels of Cu, Zn, and Cd can reach 29.7, 658, and 125.8 mg/kg, respectively [15]. Some studies have tested the metal content in the pore water of contaminated soils, where the highest levels of Cu, Zn, and Cd were found to be 6841, 39,000, and 302 mg/kg, respectively [16]. Many studies have shown that Cu, Zn, and Cd can affect plants [17,18,19,20].

Different plants have different tolerances for metals. Metal tolerance depends on the properties of the plant itself. Endophytes can stimulate plant growth and improve plant tolerance to metals through the release of small organic acids and specific ligands for metals [21]. Endophytes exhibit a mutualistic connection between plants and microorganisms. Endophytes include bacteria, fungi, and actinomycetes, which are present in plants in an asymptomatic manner for at least one life cycle [22]. Endophytes not only are valuable biological resources but also play a critical role in nature by promoting plant growth, improving plant developmental processes, enhancing stress resistance, and improving environmental adaptability [23]. Endophytes can help plants cope with stress and may also cause pathogenicity when plants face stress [24]. Endophytes increase the tolerance of host plants to drought, high temperature, heavy metal toxicity, low pH, and high salinity [25,26]. They may even confer specific disease resistance to plants [27], which has significant ecological significance for plants. The influence that endophytes have on plants stems from the constant and intricate connections they form with the host plant and other members of the host microbiome. Many studies have implanted microorganisms with high metal tolerance into plants to increase their metal tolerance [28,29]. Endophytes may also cause diseases in plants under heavy metal stress. Heavy metals can cause plant peroxidation and the production of reactive oxygen species, which can lead to changes in the role of endophytes in plants in response to these changes [30].

Several studies have shown that some endophytes can assist host plants in adapting to polluted environments [31,32]. To date, there have been no reports on how CES endophytes react to heavy metal stress. High-throughput sequencing technology was employed for the first time to analyze the species composition and diversity indices of endophytic bacteria and fungi in CES tubers under heavy metal stress. Predictions of the community functions of these endophytic organisms were made. This study has improved our understanding of how plants cope with heavy metals.

## 2. Methods and Materials

### 2.1. Plants and Culture Conditions

**Figure 1:**
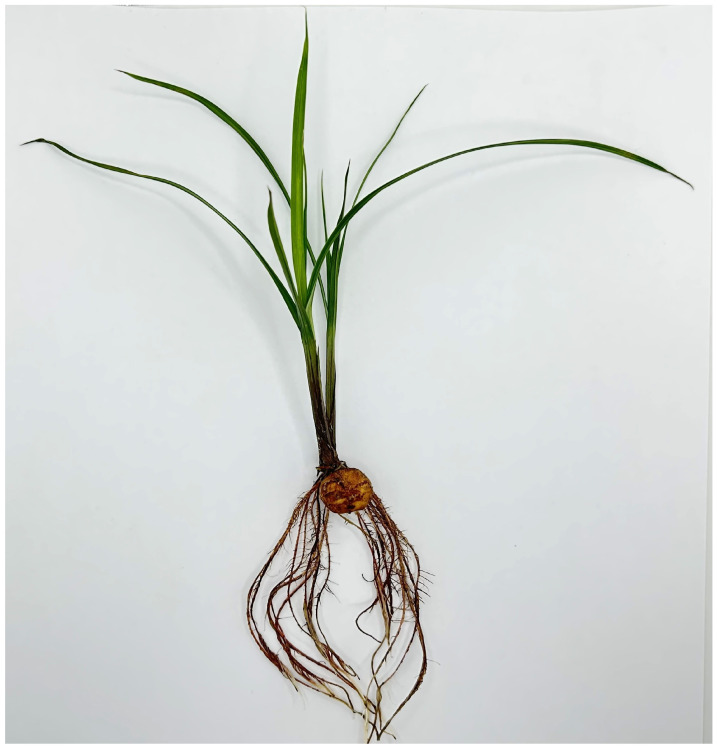
Photos of the tubers and the entire plant of *Cyperus esculentus* var. *sativus*.

Tubers of *Cyperus esculentus* var. *sativus* (CES) were collected from Baoding, Hebei, China. After the CES tubers were washed, they were soaked in 0.5% sodium hypochlorite for 30 min [33] and then placed in sterile, pure water. The tubers of CES in pure water were placed in a plant incubator at 30 °C, 70% relative humidity, and 0 lx light for seed germination. After the plants had started to grow their roots (60 h), they were placed in pure water containing various concentrations of metals for cultivation. The incubator was set up in two stages. The culture conditions in the first stage were as follows: temperature of 30 °C and relative humidity of 70%. The light conditions were 8000 lx for 14 h. The culture conditions in the second stage were as follows: the temperature was 30 °C, the relative humidity was 70%, and the light conditions were 0 lx for 10 h (cultivated in dark conditions). The initial solution volume was 700 mL. During culture, the solution was supplemented with Hoagland’s nutrient mixture to reach a volume of 700 mL. The metals used were copper (Cu), zinc (Zn), and cadmium (Cd). The samples were categorized into three treatment groups on the basis of the heavy metal concentration: 0, 1, and 5 mg/L. All three metals were combined in equal concentrations in the culture medium and were chlorinated. Each treatment group consisted of three replicates, with each replicate containing nine CES tubers. Following a 15-day cultivation period, the plants were rinsed with normal saline, dried, and subsequently stored (Figure 1).

### 2.2. Determination of Metal Element Contents in Tubers

After the samples were digested, the concentrations of copper (Cu), zinc (Zn), and cadmium (Cd) in the three tissue samples were determined via inductively coupled plasma (ICP) spectroscopy (Agilent 5110, Agilent Technologies, Inc., Santa Clara, CA, USA) [34]. Digestion: Predigestion at 120 °C for half an hour, followed by microwave digestion. The program was as follows: 130 °C, heating time 5 min, stable time 3 min; 150 °C, heating time 3 min, stable time 10 min; and 180 °C, heating time 3 min, stable time 30 min, followed by cooling to 60 °C and placing an acid catcher to catch the acid. After the acid was removed, the volume was adjusted to 25 mL with 1% nitric acid, and the mixture was shaken well for measurement. The working conditions for the examination of the elements by inductively coupled plasma emission spectrometry were as follows: the RF generator power was set to 1250 W, the plasma torch cooling gas flow rate was 15.0 L/min, the nebulizer flow rate was 0.7 L/min, the pump speed was 60 r/min, the flow rate was 1.0 L/min, the stabilization time was 20 s, and the exposure time was 5 s. On the basis of the metal content in each tissue, the translocation factor (TF) between the tissues and the bioaccumulation factor (BAF) between the CES and the environment were calculated.

### 2.3. Genomic DNA Extraction

To investigate changes in the endophytes of the CES tubers under mixed metal stress, the diversity of endophytes (endophytic bacteria and endophytic fungi) in the tubers was analyzed. DNA extraction was performed on the nine samples, and the extracted DNA was subsequently subjected to 16S rRNA and ITS sequencing. Using the CTAB method [35], total genomic DNA was extracted from the CES tubers. The extracted DNA was run on a 1% (*w*/*v*) agarose gel to assess its quality [10].

### 2.4. PCR Amplification

The DNA extracted in the previous step was subjected to the PCR amplification of the ITS1-1F region. The total DNA from the above samples, the ITS primers ITS1F (CTTGGTCATTTAGAGGAAGTAA) and ITS2 (GCTGCGTCTTCATCGATGC), a dNTP mixture, buffer, MgCl2, and Taq DNA polymerase were mixed with the appropriate amount of nucleic acid-free pure water for PCR amplification. Initially, a denaturation step was carried out at 95 °C for 3 min. The cycle amplification process was subsequently repeated, consisting of denaturation at 95 °C for 30 s, annealing at 50–60 °C for 30 s, and extension at 72 °C for 30 s to 2 min [31]. This cycle was repeated a total of 30 times. In conclusion, a final extension step was carried out for 5–10 min at 72 °C, followed by the analysis and purification of the PCR products via agarose gel electrophoresis. The previous primers were replaced with the 16S rRNA primers 799F (AACMGGATTAGATACCCKG) and 1193R (ACGTCCCCACCTTCC) to amplify the 16S V57 region of the DNA extracted in the previous step [36]. The rest of the steps were the same.

### 2.5. Library Preparation, Sequencing, and Raw Data Processing

The construction of the library was carried out via the NEBNext^®^ Ultra™ II DNA Library Prep Kit (New England Biolabs (NEB), Ipswich, MA, USA). The constructed library was subjected to Qubit and qPCR quantification, with the index code added during the process. The library quality was subsequently examined with a Qubit 2.0 fluorescence instrument (Thermo Fisher Scientific Inc., Waltham, MA, USA) and an Agilent Bioanalyzer 2100 system. After the library was qualified, it was subjected to paired-end sequencing (Paired-End) via the Illumina NovaSeq sequencing platform to generate 200–400 bp paired-end reads. The reads were identified on the basis of their respective barcodes, and then the barcodes and primer sequences were removed. FLASH v1.2.7 [37] was used to merge the paired-end reads. Following the quality control steps in QIIME V1.9.1 [38], the sequences were subsequently aligned with the Silva reference database for comparison [39]. Following a comparison, chimeric sequences were detected and then eliminated from the dataset. Only when the reads carried the correct barcode and forward primer sequence, had an average quality score of ≥25, and had a length in the range of 200–400 bp were the reads retained. After the processing outlined above, the raw data were collected. The original sequencing data contained a certain proportion of dirty data. To guarantee the precision and dependability of the findings from the data analysis, the raw data were initially spliced and filtered to acquire clean data. DADA2 [40] performs noise reduction and filters out sequences with an abundance of less than 5 [41] to obtain the final amplicon sequence variants (ASVs) [42].

### 2.6. Species Composition Analysis

The DADA2 method is more sensitive and specific than traditional OTU methods and is able to detect real biological variants that are missed by OTU methods while outputting fewer pseudosequences [43]. Moreover, the substitution of OTUs with ASVs increased the accuracy, comprehensiveness, and reproducibility of marker gene data analysis [44]. Using the classify-sklearn algorithm of QIIME2 [45,46], the pretrained naive Bayes classifier was used for species annotation for each ASV. After the ASV annotations and the characteristic table of each sample were analyzed, a species abundance table was generated at the order, family, and genus levels for the analysis of species composition and differences between samples, cluster analysis, etc.

### 2.7. α Diversity and β Diversity Analysis

The diversity of the microbial community in the sample was examined via alpha diversity [47]. The richness and diversity of the microbial community in a sample can be reflected through single-sample diversity analysis. This includes the use of a series of statistical analysis indices, such as the observed species, Shannon, Simpson, Chao1, Goods_coverage, and Pielou_e indices. Furthermore, the species richness and diversity among each sample were compared via a species diversity curve and a dilution curve.

Beta diversity was determined through a comparative analysis of the microbial community composition in different samples. First, using the species annotation results and the abundance information of the ASVs for all the samples, the ASVs with the same classification were merged and processed to obtain a table of species abundance information (Profiling Table). Moreover, the phylogenetic relationships among the ASVs were used to further calculate the unifrac distance (Unweighted unifrac) [48,49]. By incorporating the evolutionary information of microbial sequences within each sample, the unifrac distance method calculates the distance between samples. In cases where there are more than two samples, a distance matrix is generated. The unifrac distance (Unweighted unifrac) was then further constructed into the weighted unifrac distance via the abundance of information on ASVs [50]. Ultimately, the beta diversity index was analyzed for differences between groups. By using multivariate statistical approaches such as PCA and NMDS, differences among various samples (groups) can be discerned.

### 2.8. Function Prediction

The obtained ITS sequencing data were analyzed against the MetaCyc database (https://metacyc.org/ (accessed on 20 September 2024)) via the FUNGuild (FUNGuild v1.0, the Swiss Federal Institute of Technology in Lausanne, Switzerland) tool [51], and the results were used to classify fungal species into different functional groups. The functional groups were classified according to the functional annotations of the fungi. We passed the obtained 16S rRNA sequencing data through PICRUSt2 (version 1.9, David J. Balding and Rodney J. Thompson, Lincoln University, Lincoln, New Zealand) for functional prediction on the basis of the KEGG, COG, PFAM, TIGRFAM, and MetaCyc databases. The relative abundance or relative expression of each functional group in each sample was obtained. Additionally, information on functional group annotation is available to provide deeper insight into the biological functions of diverse functional groups present in the CES endophyte community.

### 2.9. Statistical Analysis

To assess the magnitude of differences between samples, statistical analyses were performed via SPSS (IBM SPSS Statistics 26) software. The *t* test was used to compare samples between two groups, and the Tukey test was used to compare samples between more than two groups. When the *p*-value was less than 0.05, a statistically significant difference between the groups was indicated.

## 3. Results

### 3.1. Metal Content in Tubers

The contents of all three metals in the tubers of the three groups of samples were significantly different. The metal contents of the tubers in the control group were 1.17 ± 0.04 μg/g for Cu, 10.71 ± 0.50 μg/g for Zn, and 1.2 ± 0.05 μg/g for Cd. The metal contents of the tubers in the group treated with low concentrations of metals were 2.65 ± 0.12 μg/g for Cu, 12.14 ± 0.43 μg/g for Zn, and 2.29 ± 0.11 μg/g for Cd. The metal contents of the tubers in the groups treated with high concentrations of metals were 4.22 ± 0.20 μg/g for Cu, 4.22 ± 0.20 μg/g for Zn, and 2.29 ± 0.11 μg/g for Cd. The tubers in the high-metal-concentration treatment group presented metal concentrations of 4.22 ± 0.20 μg/g for Cu, 17.89 ± 0.63 μg/g for Zn, and 4.67 ± 0.17 μg/g for Cd. An increase in the metal concentration led to greater metal accumulation in the tubers.

### 3.2. Quality Control and Processing of Sequencing Data

**Figure 2:**
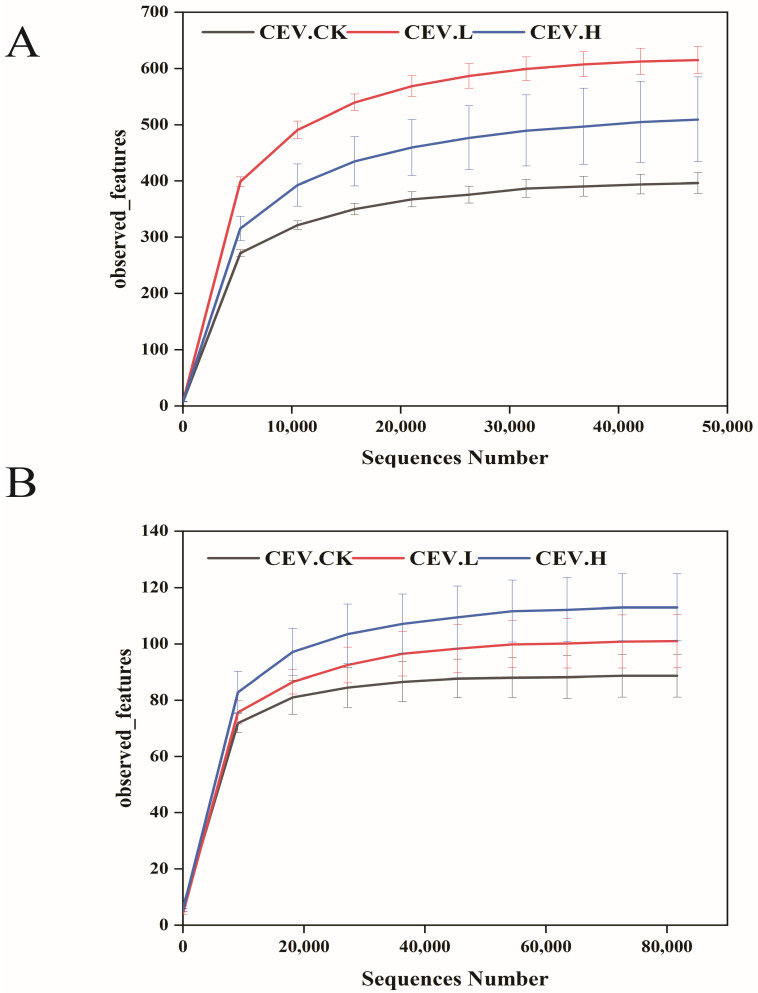
Sparsity plot for each sample based on the observed_features index. Note: (**A**): endophytic bacteria; (**B**): endophytic fungi. cev.ck: no added metal; cev.l: metal concentration of 1 mg/L; cev.h: metal concentration of 5 mg/L.

Using ITS (endophytic fungi) and 16S rRNA (endophytic bacteria) high-throughput sequencing technologies, fungal and bacterial diversity analyses of the endophytes of CES tubers under different metal stresses were conducted. As shown in Figure 2A, the sparsity curves of the observed features of endophytic bacteria in the different samples revealed that as the number of sequencing reads increased, the number of observed species gradually increased. When the number of sequenced reads approached 40,000, the curve tended to flatten, indicating that the sequencing reading was sufficient to reflect the overall community structure of the endophytic bacteria in the sample. There were approximately 600 observed species. The average number of clean reads per sample for the subsequent analysis was 70,603 after chimeras, low-quality sequences, and short sequences were eliminated. As depicted in Figure 2B, the sparsity curves for the detected features of endophytic fungi across various samples indicated that with a rise in the quantity of sequencing reads, there was a gradual increase in the number of species observed. When the number of sequenced reads approached 60,000, the curve tended to flatten, indicating that the sequencing reading was sufficient to reflect the overall community structure of the endophytic fungi. There were approximately 100 observed species. Following the elimination of chimeras, low-quality sequences, and short sequences, the mean number of clean reads per sample available for the subsequent analysis was 90,708.

### 3.3. Species Composition Analysis

**Figure 3:**
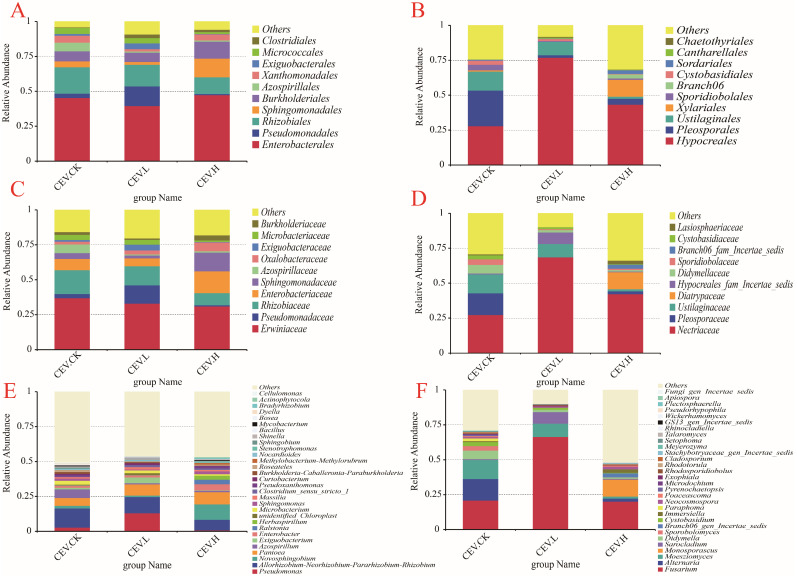
Species composition of tuber endophytes under different concentrations of metals. Note: CEV. CK: no added metal; CEV. L: metal concentration of 1 mg/L; CEV. H: metal concentration of 5 mg/L. (**A**): endophytic bacterial composition of the order, (**B**): endophytic fungal composition of the order, (**C**): endophytic bacterial composition of the family, (**D**): endophytic fungal composition of the family, (**E**): endophytic bacterial composition of the genus, and (**F**): endophytic fungal composition of the genus.

The reads were counted, and the composition distribution of each sample at the order, family, and genus levels was visualized via QIIME2 (2019.4). At the order level, we compared the ten most abundant orders in the different samples. Figure 3A shows the species composition of the order of endophytic bacteria. The bacterial orders with relatively high abundances in all of the samples were Enterobacterales, Pseudomonadales, Rhizobiales, Sphingomonadales, and Burkholderiales. Enterobacterales was the most abundant bacterial order among all of the samples. Compared with those in the control group, the proportions of Pseudomonadales and Exiguobacterales in the low-concentration metal treatment group increased by 10.97% and 2.95%, respectively, whereas the proportions of the other orders decreased. Compared with those in the control group, the proportions of Sphingomonadales and Burkholderiales in the high-concentration metal treatment group increased by 9.12% and 5.00%, respectively. Figure 3B shows the species composition of the order of endophytic fungi. The orders with relatively high abundance in all of the samples were Hypocreales, Pleosporales, and Ustilaginales. Hyporeales was the most abundant fungal order among all the samples. Compared with those in the control group, the proportions of Hypocreales in the low-concentration and high-concentration metal treatment groups increased by 48.92% and 15.37%, respectively; the proportions of Pleosporales decreased by 23.65% and 21.26%, respectively; and the proportions of Ustilaginales decreased by 4.04% and 12.11%, respectively.

At the family level, the ten most abundant families in different samples were compared. Figure 3C shows the species composition of the family of endophytic bacteria. The families with relatively high abundances in all of the samples were Erwiniaceae, Pseudomonadaceae, Rhizobiaceae, Enterobacteriaceae, and Sphingomonadaceae. Erwiniaceae was the most abundant bacterial family in all of the samples. Compared with those in the control group, the proportion of Pseudomonadaceae in the low-concentration metal treatment group increased by 10.06%. Additionally, the proportions of Rhizobiaceae, Enterobacteriaceae, and Sphingomonadaceae decreased by 3.40%, 2.31%, and 2.28%, respectively. The proportions of Pseudomonadaceae and Rhizobiaceae decreased by 2.18% and 8.36%, respectively, and the proportions of Enterobacteriaceae and Sphingomonadaceae increased by 7.69% and 9.12%, respectively, in the high-concentration metal-treated group compared with those in the control group. Figure 3D shows the species composition of the family of endophytic fungi. The families with relatively high abundances in all of the samples were Nectriaceae, Pleosporaceae, and Ustilaginaceae. Nectriaceae was the most abundant fungal family in all of the samples. Compared with those in the control group, the proportion of Nectriaceae in the low-concentration metal treatment group increased by 41.23%, whereas the proportions of Pleosporaceae and Ustilaginaceae decreased by 15.46% and 4.04%, respectively. Compared with those in the control group, the proportion of Nectriaceae in the high-concentration metal treatment group increased by 14.85%, whereas the proportions of Pleosporaceae and Ustilaginaceae decreased by 13.33% and 12.11%, respectively. The proportion of Diatrypaceae in the high-concentration metal treatment group was 12.12%, whereas this type of fungus was not found in the control group or the low-concentration metal treatment group.

At the genus level, the 30 most abundant families in different samples were compared. Figure 3E shows the species composition of the genera of endophytic bacteria. The genera with high abundance in all of the samples were *Pseudomonas*, *Allorhizobium-Neorhizobium-Parrhizobium-Rhizobium*, *Novosphingobium*, *Pantoea*, *and Azospirillum*. Compared with those in the control group, the proportions of *Pseudomonas* and *Pantoea* increased by 10.27% and 2.36%, respectively. The proportions of *Allorhizobium-Neorhizobium-Pararhizobium-Rhizobium* and *Azospirillum* decreased by 2.04% and 5.38%, respectively. Compared with those in the control group, the proportions of *Novosphingobium* and *Pantoea* increased by 9.19% and 3.17%, respectively. The proportions of *Allorhizobium-Neorhizobium-Pararhizobium-Rhizobium* and *Azospirillum* decreased by 6.16% and 5.30%, respectively. The proportion of *Actinophytocola* in the high-concentration treatment group was 0.65%, while this type of bacteria was not found in the control group or low-concentration treatment group. Figure 3F shows the species composition of the genera of endophytic fungi. The genera with the greatest abundance in all of the samples were *Fusarium*, *Alternaria*, *Moesziomyces*, *Monosporascus*, and *Sarocladium*. *Fusarium* was the most abundant fungal genus among all of the samples. Compared with those in the control group, the proportions of *Fusarium* and *Sarocladium* in the low-concentration metal treatment group increased by 45.55% and 7.70%, respectively. The proportions of *Alternaria*, *Moesziomyces*, and *Didymella* decreased by 15.46%, 4.04%, and 4.52%, respectively. Compared with those in the control group, the proportions of *Alternaria* and *Moesziomyces* in the high-concentration metal treatment group decreased by 13.33% and 12.11%, respectively. The proportion of *Monosporascus* in the high-concentration metal treatment group was 12.12%, whereas this type of fungus was not found in the control group or the low-concentration metal treatment group.

### 3.4. α Diversity

**Figure 4:**
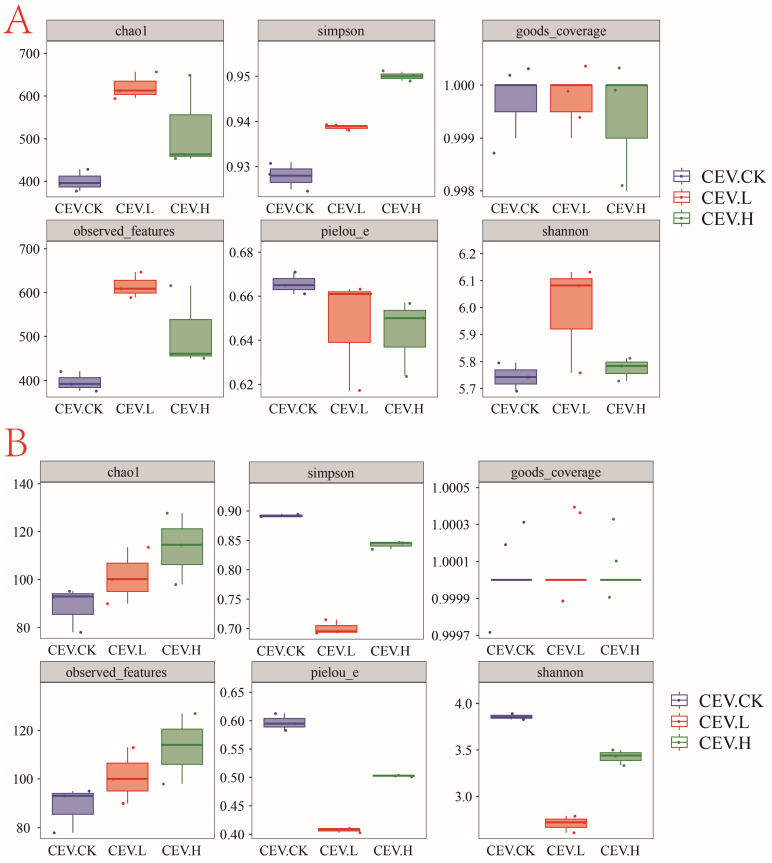
α diversity of tuber endophytes. Note: CEV. CK: no added metal; CEV. L: metal concentration of 1 mg/L; CEV. H: metal concentration of 5 mg/L. (**A**): endophytic bacteria; (**B**): endophytic fungi.

The values of α diversity, including the Chao1, Simpson, Good’s coverage, observed feature, Pielou’s E, and Shannon indices, were used to analyze the species diversity and distribution of endophytes in the tubers under different treatments. The alpha diversity indices of the endophytic bacteria are shown in Figure 4A. Compared with those of the control group, the Chao1, observed feature, and Shannon indices of the low-concentration metal treatment group were significantly greater. The Simpson index continued to increase significantly with increasing metal concentration. Compared with that in the control group, the Pielou_e index in the low- and high-metal concentration treatment groups decreased, but this difference did not reach statistical significance. The alpha diversity indices of the endophytic fungi are shown in Figure 4B. Compared with those in the control group, significant decreases in the Pielou_e, Shannon, and Simpson indices and significant increases in the observed_features and Chao1 indices occurred in the low-concentration metal treatment group. Compared with those in the control group, the Pielou_e, Shannon, and Simpson indices were significantly lower, whereas the observed_features and Chao1 indices were significantly greater in the high metal concentration group. Compared with those in the low-metal treatment group, the Pielou_e, Shannon, and Simpson indices significantly increased, whereas the observed_features and Chao1 indices did not significantly increase in the high-metal treatment group. The Good’s coverage indices of the three groups of samples are all 1.

### 3.5. β Diversity

**Figure 5:**
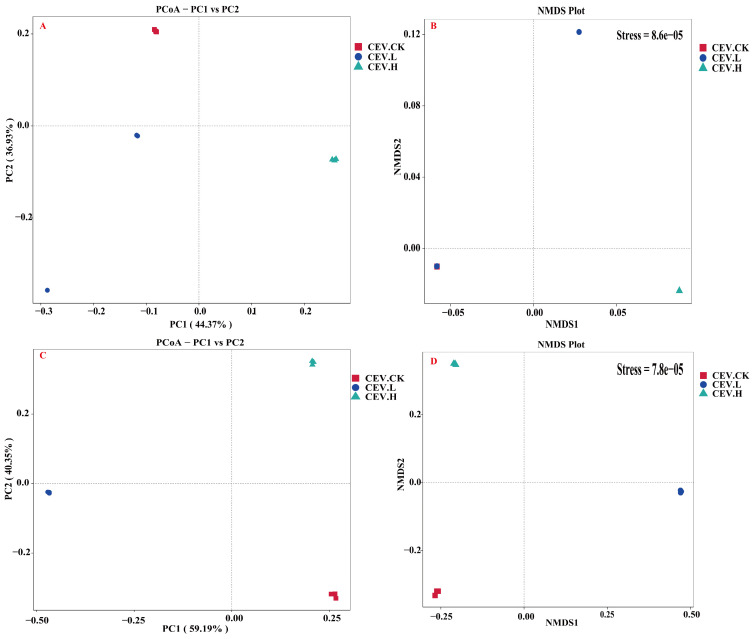
NMDS and PCoA of tuber endophytes. Note: CEV. CK: no metal added; CEV. L: metal concentration of 1 mg/L; CEV. H: metal concentration of 5 mg/L. (**A**): PCoA of endophytic bacteria. (**B**): NMDS analysis of endophytic bacteria. (**C**): PCoA of endophytic fungi. (**D**): NMDS analysis of endophytic fungi.

We employed the Bray–Curtis dissimilarity distance matrix to conduct a principal coordinate analysis (PCoA) and nonmetric multidimensional scaling (NMDS) to assess community variation among the three sample groups. This allowed us to assess the effects of metals on the endophytes of oilseed bean tubers. The β diversity of the endophytic bacteria is shown in Figure 5A,B. The results of the PCoA and NMDS analysis revealed that compared with those in the control group, the endophytic bacterial community structure in tubers in the low-concentration metal treatment and high-concentration metal treatment groups changed. The endophytic bacterial community structures of the low-concentration treatment group and the high-concentration treatment group also tended to differ. The β diversity of the endophytic fungi is shown in Figure 5C,D. The results of the PCoA and NMDS analysis revealed that compared with that in the control tubers, the community structure of endophytic fungi in the tubers in the low-concentration and high-concentration metal treatment groups changed. Similarly, the endophytic fungal community structure tended to differ between the low-concentration treatment group and the high-concentration treatment group.

**Figure 6:**
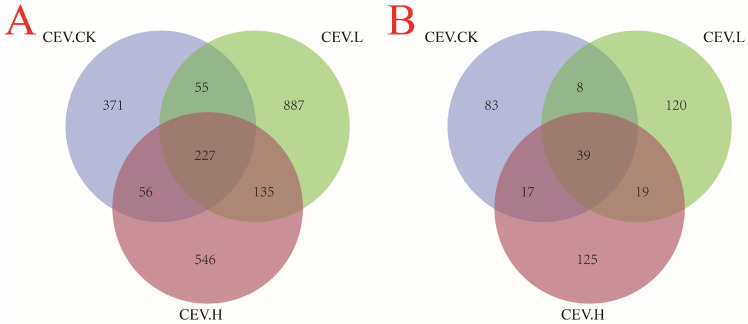
Venn diagram of the ASVs of the CES endophytes. Note: CEV. CK: no added metal; CEV. L: metal concentration of 1 mg/L; CEV. H: metal concentration of 5 mg/L. (**A**): endophytic bacteria; (**B**): endophytic fungi.

To more clearly show the differences in species communities among the three groups of samples, we constructed a Venn diagram to display the unique and common ASVs among the samples, as shown in Figure 6. Figure 6A shows the Venn diagram of endophytic bacteria in the three groups of samples. There were 709 ASVs in the control group, including 371 unique ASVs, 282 ASVs shared with the low-concentration metal treatment group, and 283 ASVs shared with the high-concentration metal treatment group. There were 1304 ASVs in the low-concentration metal treatment group, including 887 unique ASVs. A total of 362 ASVs were shared by the high-concentration metal treatment group. There were 964 ASVs in the high-concentration metal treatment group, with 546 unique ASVs. Figure 6B shows the Venn diagrams of the endophytic fungi in the three groups of samples. There were 147 ASVs in the control group, among which 83 were unique, 47 were shared with the low-concentration metal treatment group, and 56 were shared with the high-concentration metal treatment group. There were 186 ASVs in the low-concentration metal treatment group, among which 120 were unique. Fifty-eight patients were included in the high-concentration metal treatment group. There were 200 ASVs in the high-concentration metal treatment group, including 125 unique ASVs.

### 3.6. Community Function Prediction

**Figure 7:**
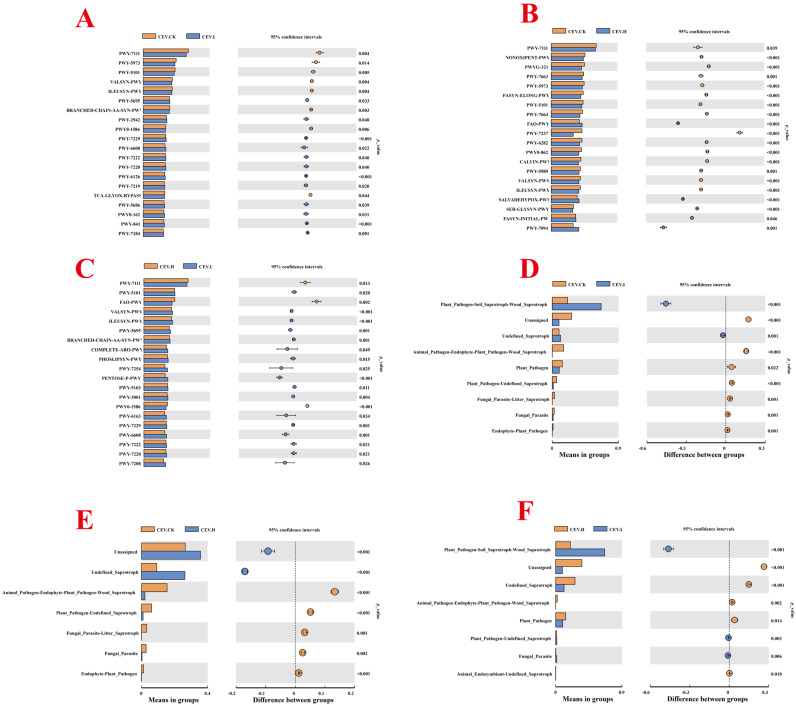
Community functional abundance of tuber endophytes between different groups (**A**–**F**). Note: CEV. CK: no added metal; CEV. L: metal concentration of 1 mg/L; CEV. H: metal concentration of 5 mg/L. The left panel displays the different functions between the groups. Each bar in the graph represents the mean abundance difference between the different subgroups. The right panel shows the confidence level of the difference between the groups. The leftmost endpoint of each circle represents the lower limit of the 95% confidence interval for the mean difference, whereas the rightmost endpoint represents the upper limit. The rightmost endpoint of each circle indicates the upper limit of the 95% confidence interval for the mean difference. The center of the circle represents the difference in means, whereas the color represents the *p*-value of the significant between-group difference test for the corresponding difference function.

**Figure 8:**
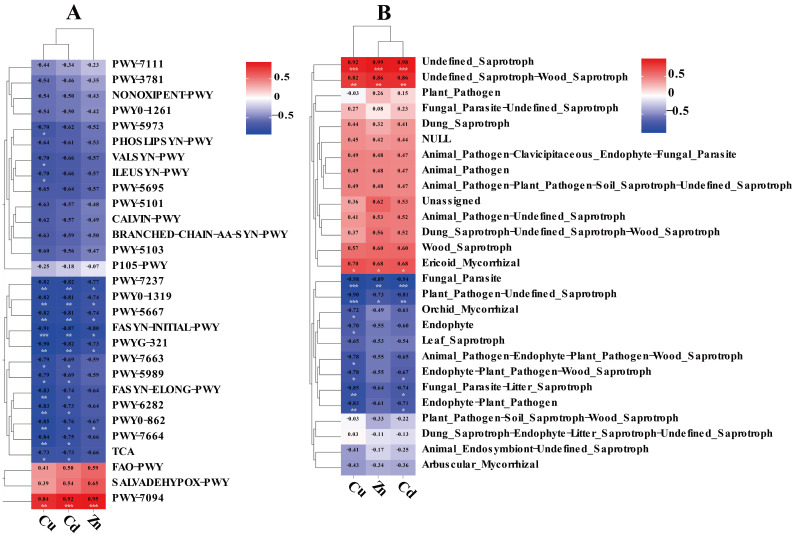
Correlation heatmap analysis between endophytic bacterial function (**A**) and endophytic fungal guilds (**B**) and tuber metal content. Note: * correlations are significant between 0.01 and 0.05, ** correlations are significant between 0.001 and 0.01, and *** Pearson’s correlation coefficient is significant at the 0.001 level.

To explore the effects of heavy metals on the function of endophytic bacteria in tubers, the PICRUSt2 (version 1.9, David J. Balding and Rodney J. Thompson, Lincoln University, New Zealand) and FUNGuild (FUNGuild v1.0, the Swiss Federal Institute of Technology in Lausanne, Switzerland) tools were used to predict the community functions of endophytes in the three groups of samples. Figure 7A–C shows the mean values of functional pathway abundance of the endophytic bacterial community in the tubers. As shown in Figure 7A, significant changes occurred in 116 metabolic pathways in the low-concentration metal treatment group compared with those in the control group. PWY-5971, PWY-5676, P381-PWY, PWY-7332, PWY-2941, PWY-5507, and PWY-6590 all significantly increased, accounting for approximately 738.06%, 187.86%, 221.06%, 431.22%, 339.65%, 300.77%, and 299.31%, respectively, of the control group. PWY-7098, P184-PWY, PWY-7097, PWY-6338, FUC-RHAMCAT-PWY, and PWY-6992 all significantly decreased, accounting for approximately 55.59%, 56.91%, 55.31%, 55.31%, 50.28%, and 53.24%, respectively, of the control group. PWY-6919 was detected in the low-concentration metal treatment group but not in the control group. As shown in Figure 7B, compared with those in the control group, 340 metabolic pathways in the high-concentration metal treatment group significantly changed. Compared with those in the control group, the abundances of PWY-5971, PWY-5420, PWY-5529, PWY-7332, CHLOROPHYLL-SYN, and PWY-5183 significantly increased and were approximately 328.20%, 244.41%, 540.62%, 302.56%, 592.83%, and 429.58%, respectively. PWY-5028, PWY-5971, PWY-3661, PWY-2941, and PWY-6992 all showed significant reductions, accounting for approximately 77.91%, 44.48%, 63.16%, 13.48%, and 50.17%, respectively, of the low-concentration metal treatment group. PWY-6993 was detected in the control group but not in the high-metal treatment group. As shown in Figure 7C, compared with those in the low-concentration metal treatment group, 144 metabolic pathways in the high-concentration metal treatment group presented significant changes. The levels of PWY-6339, PWY-6891, PWY-7098, P184-PWY, and PWY-5419 increased significantly and were approximately 234.72%, 203.34%, 339.60%, 320.94%, and 402.07%, respectively, of those in the low-concentration metal treatment group. PWY-5028, PWY-5971, PWY-3661, PWY-2941, and PWY-6992 all decreased significantly, by approximately 77.91%, 44.48%, 63.16%, 13.48%, and 50.17%, respectively, in the low-concentration metal treatment group. Figure 8A shows the Pearson correlation heatmap of the contents of the three metals in tubers and the abundance of the functional pathways of endophytic bacteria. The three metals were significantly negatively correlated with the abundance of the functional pathways associated with most endophytic bacteria in the tubers. The zinc content was significantly correlated with seven pathways, whereas the copper content was significantly correlated with sixteen pathways. The abundance of PWY-7094 (fatty acid salvage) was strongly positively correlated with the contents of all three metals.

Figure 7D–F shows the mean values of the functional abundance of endophytic fungi in the tubers. As shown in Figure 7D, compared with those in the control group, the percentages of plant_Pathogen-Soil_Saprotroph-Wood_Saprotroph and Undefined_Saprotroph significantly increased in the low-concentration metal-treated groups; these percentages were approximately 318.28% and 123.79%, respectively, of those in the control group. Compared with those of the control group, the percentages of the animal_pathogen-endophyte-plant_pathogen-wood_saprotroph, plant_pathogen, plant_pathogen-undefined_saprotroph, fungal_parasite-litter_saprotroph, fungal_parasite, and endophyte-plant_pathogen groups were significantly lower, at approximately 0.22%, 69.03%, 26.44%, 1.14%, 48.59%, and 2.78%, respectively. Orid_Mycorrhizal and Endophyte-Plant_Pathogen-Wood_Saprotroph were not found in the low-concentration metal treatment group. As shown in Figure 7E, compared with that in the control group, the number of Undefined_Saprotrophs was significantly greater in the high-concentration metal-treated group, which was approximately 284.80% greater than that in the control group. The numbers of Animal_Pathogen-Endophyte-Plant_Pathogen-Wood_Saprotroph, Plant_Pathogen-Undefined_Saprotroph, Fungal_Parasite-Litter_Saprotroph, Fungal_Parasite, Endophyte-Plant_Pathogen, Orchid_Mycorrhizal, and Endophyte-Pathogen-Wood_Saprotrophs were significantly lower, approximately 13.94%, 16.54%, 1.52%, 12.78%, 7.69%, 15.51%, and 3.55%, respectively, than those in the control. As shown in Figure 7F, compared with those in the low-concentration metal treatment group, Undefined_Saprotroph, Animal_Pathogen-Endophyte-Plant_Pathogen-Wood_Saprotroph, Plant_Pathogen, and Animal_Endosiont-Undefined_Saprotroph significantly increased by approximately 230.06%, 6298.81%, 142.44%, and 201.19%, respectively, in the low-concentration metal treatment groups. Plant_Pathogen-Soil_Saprotroph-Wood_Saprotroph Plant_Pathogen-Soil_Salprotroph-Wood_Saprotroph, Plant_Pathogen-Undefined_Saprotroph, and Fungal_Parasite significantly decreased by approximately 30.32%, 62.58%, and 26.30%, respectively, in the low-concentration metal treatment groups. Figure 8B shows the heatmap of the Pearson correlation between the contents of the three metals in the tubers and the abundance of the functional pathways of endophytic fungi. The zinc content was significantly correlated with only five functional pathways, whereas the copper content was significantly correlated with eleven pathways. Two undefined functional pathways of saprophytes exhibited strong positive correlations with the contents of the three metals. The contents of the three metals were strongly negatively correlated with the abundance of Fungal_Parasite.

## 4. Discussion

Heavy metals in the environment, especially nonessential metals such as cadmium, can strongly damage plant tissues. At certain concentrations, essential metals such as copper and zinc can also cause damage to plant tissues [52]. Common damage to plants from metals is lipid peroxidation and protein destruction or degradation, and metals also damage processes such as photosynthesis and respiration in plants [53]. The results revealed that the number of species of tuber endophytic bacteria and fungi increased with the addition of mixed metals. The α diversity and β diversity indices of endophytes in the tuber also changed significantly with the addition of mixed metals. The changes in the diversity indices of tubers subjected to different concentrations of mixed metals indicate that the diversity of endophytic fungi and endophytic bacteria in tubers respond differently to mixed metals. Metals reduce microbial diversity in the environment [54]. In this study, the number of endophytic bacteria and endophytic fungal species in the CES tubers increased. Even the abundance of the endophytic bacteria Shannon, Simpson, and Chao1 significantly increased. This may be because there are many species with metal tolerance in the CES tubers, but these species do not exhibit metal tolerance because of the inhibition of interspecific competition. Metal treatment led to the dominance of these metal-tolerant species [55]. The greater effect of low concentrations of mixed metals on tuber endophyte diversity may be because high concentrations of mixed metals suppressed the physiological functions of CES to a greater extent and indirectly inhibited the ability of CES to reassemble the endophyte flora.

The species composition of CES tuber endophytes varied with the addition of mixed metals. Low concentrations of mixed metals can increase the abundance of *Pseudomonas*, making it the dominant endophytic bacterial genus in tubers. Multiple studies have shown that Pseudomonas can improve the utilization of nutrients in the environment, change the secretion of host organic acids, and increase the host plant’s metal uptake ability [56,57]. The present study also revealed significant changes in the pathways related to organic acid metabolism, which may imply that *Pseudomonas* has similar potential in CES tubers in response to metal stress. High concentrations of mixed metals significantly increased the abundance of *Novosphingobium*, making *Novosphingobium* the dominant endophytic bacterial genus in the tubers. Studies have reported that *Novosphingobium* is tolerant to metals and promotes plant growth through the secretion of auxins such as IAA [58]. We identified a novel endophytic bacterial genus, *Actinophytocola*, at high concentrations in the mixed-metal tubers. A low concentration of mixed metals greatly increased the abundance of *Fusarium*. Multiple studies have shown that *Fusarium* promotes plant growth through the production of IAA and ACCD and is tolerant to various heavy metals, such as copper, zinc, and cadmium [59,60]. In this study, *Fusarium* was also found in the control tubers, and we did not observe significant symptoms in the control tubers. *Fusarium* has been used in the phytoremediation of plant diseases in some studies [61]. *Fusarium* has good metal resistance, and the metal tolerance of *F. oxysporum* may be attributed to the production of selected antioxidant enzymes and organic acids in response to metal pressing [62]. The abundances of *Alternaria* and *Moesziomyces* were significantly reduced, indicating that these fungi have low tolerance to mixed metals. Multiple studies have reported that *Alternaria* and *Moesziomyces* can cause various plant diseases, such as leaf spot, fusarium wilt, and smut. Various species of Alternaria can even cause plant necrosis [63,64,65]. *Monosporascus* was found only in the high-concentration metal treatment group. It is possible that *Monosporascus* has greater adaptability to mixed metals. Moreover, high concentrations of mixed metals inhibited the growth of other endophytic fungi and weakened competition for *Monosporascus*. We also observed that low concentrations of metals can increase the abundance of rhizobia, which can absorb nitrogen from the environment for host utilization and promote plant growth [66,67]. Our findings combined with those of the existing studies suggest that CES tubers reassemble the species composition of endophytes to adapt to the combined stresses of Cu, Zn, and Cd.

Changes in the species composition of CES tuber endophytes affect endophyte community function and the relationships between endophytes and CES interactions. Significant changes in the metabolic pathways related to amino acid metabolism, the TCA cycle, sugar metabolism, and fatty acid metabolism were observed in the endophytic bacteria of tubers. Our study demonstrated that the functional abundance of multiple saprophytic relationships in the tuber endophytic fungal community was elevated and that the functional abundance of the parasitic fungal relationships was significantly reduced. This implies that the interaction relationship between the tuber endophytic fungi and CES was altered to adapt to the mixed metal stress. Significant changes occurred in multiple metabolic pathways related to isoleucine, valine, and lysine in the endophytes of the tubers. These amino acids can be used as raw materials for many metal-chelating agents, such as glutathione [68]. Chelating agents can help plants absorb essential metals from the environment and balance the concentration of metals in plants [69]. The TCA cycle is an important energy metabolism process [70]. Some studies have reported that endophytes can change enzyme activities related to respiration and photosynthesis in host plants [71] and affect the synthesis of metabolites such as acetyl-CoA and pyruvate in plants [72]. The TCA cycle plays a crucial role in defending against oxidative stress and reducing the toxicity of metals to cells [73]. PWY-711 (pyruvate fermentation to isobutanol) is the most abundant metabolic pathway in tuber endophytes. Isobutanol is a relatively high-value biofuel [74]. It has been reported that endophytes in plants can produce isobutanol [75,76], including the highly abundant Pseudomonas in this study [77]. Isobutanol has certain antioxidant effects [78]. CES has some antioxidant capacity [79], and the most common damage caused by metals to organisms is oxidative damage. Therefore, we hypothesize that isobutanol is an important compound for endophytes to assist CES in coping with metal stress. Metals can induce lipid peroxidation in the cell membrane, which affects fatty acid levels [80]. In the present study, the PWY-7094 (fatty acid salvage) pathway was strongly and positively correlated with the levels of the three metals, suggesting that endophytes may attenuate the damage caused by metals to cell membranes by increasing the fatty acid content. Changes in tuber endophyte community function and endophyte–CES interactions help CESs cope with and adapt to metal stress [36].

## 5. Conclusions

Under mixed heavy metal stress, the species composition of endophytic microorganisms in the tubers changed, with the microorganisms exhibiting greater tolerance to mixed metals assuming dominance. The diversity of endophytic bacteria in the tubers increased, indicating a shift in community structure and composition. The reassembled community of endophytic microorganisms in the tubers helped the host plant, *Cyperus esculentus* var. *sativus* (CES), better adapt to heavy metal stress. Mixtures of heavy metals at certain concentrations promoted the growth of beneficial microorganisms for plant growth in the CES tubers while inhibiting the growth of harmful microorganisms. CES adapts to heavy metal stress by quickly adjusting the composition of endophyte species. This study has advanced our understanding of the adaptation mechanisms of heavy metals in plants.

## Data Availability

All the data analyzed during this study are included in this article.
